# Infectious Disease: New Human Retroviruses

**DOI:** 10.1289/ehp.113-a810a

**Published:** 2005-12

**Authors:** Melissa Lee Phillips

Retroviruses called human T-lymphotropic viruses (HTLVs) are found in two types—HTLV-1 and HTLV-2—in people all over the world. Genetic evidence suggests that they crossed into humans from simian T-lymphotropic viruses (STLVs) and that each type, plus various subtypes, have crossed independently. Now, two more types of HTLV have been found in humans living in central Africa.

At least 22 million humans are infected with HTLV-1 or HTLV-2, and the viruses are endemic in several areas. About 2–5% of those infected with HTLV-1 develop adult T cell leukemia. HTLV-1 also causes a neurologic disease called tropical spastic paraparesis/HTLV-1 associated myelopathy. HTLV-2 is less pathogenic but is thought to cause similar neurologic illnesses and increase susceptibility to opportunistic infection.

William Switzer, a researcher at the Centers for Disease Control and Prevention, and his colleagues sequenced HTLV strains from a high-risk population: people in Cameroon who reported contact with nonhuman primate tissues through hunting and butchering or keeping primate pets. The study uncovered many previously unknown subtypes of HTLV-1, most with known correlates in nonhuman primates. The team also found that two people carried previously unknown HTLV types. One, HTLV-3, is similar to the nonhuman primate virus STLV-3. The other, HTLV-4, is genetically different from any known virus in humans or other primates. The findings appear in the 31 May 2005 issue of the *Proceedings of the National Academy of Sciences*.

Because HTLV-4 is so divergent from other HTLVs, this virus may have evolved in humans over quite some time, Switzer says. It’s possible, though, that primates are infected with an equally divergent simian version that just hasn’t been found yet. “We’re screening primates in that same area to see if we can answer that question,” Switzer says.

A group led by Antoine Gessain, head of the Epidemiology and Physiopathology of Oncogenic Viruses Unit at the Pasteur Institute, also recently found a subtype of HTLV-3 in a human, but it’s somewhat different from the subtype Switzer and his colleagues found, which suggests “another example of multiple independent, cross-species transmission events,” Switzer says. The HTLV-3 strain Gessain found is extremely similar to a strain reported in the red-capped mangabey, which suggests that it crossed to humans very recently, Switzer says. Gessain’s findings were published 9 May 2005 in *Retrovirology*.

The current dogma surrounding retroviruses is that cross-species transmission is rare, but finding so many near-identical strains between humans and nonhuman primates suggests this is not a rare event. Benign retroviruses probably cross from nonhuman primates to humans frequently, but we don’t notice them because we don’t get sick, says Bernard Poiesz, a professor of medicine at SUNY Upstate Medical University. “But every once in a while,” he says, “one of them will jump and we may not handle it so well.”

## Figures and Tables

**Figure:**
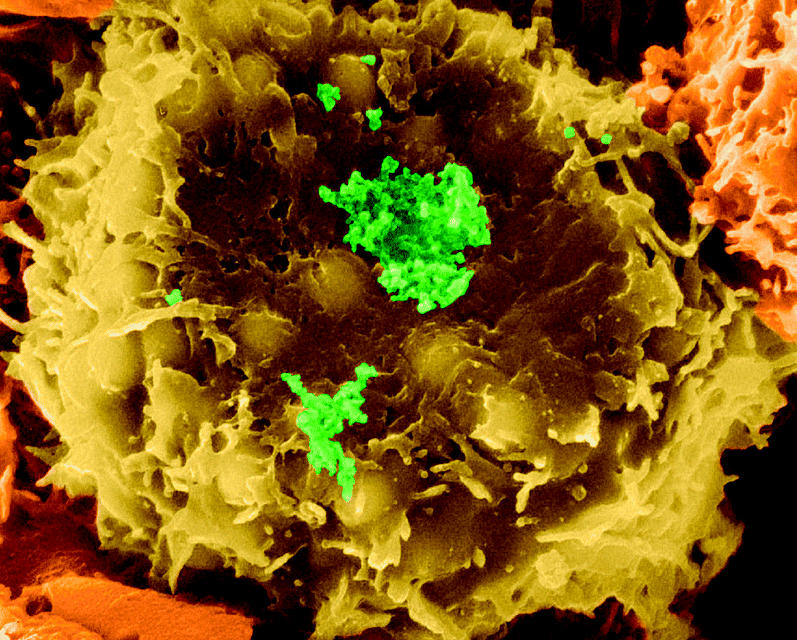
Cross-species predator. A T lymphocyte infected with HTLV-1 (green), which causes a type of leukemia. Such viruses are believed to have crossed to humans from simians.

